# Glutathione and catalase suppress TGFβ-induced cataract-related changes in cultured rat lenses and lens epithelial explants

**Published:** 2009-05-01

**Authors:** Coral G. Chamberlain, Kylie J. Mansfield, Anna Cerra

**Affiliations:** School of Medical Sciences (Anatomy and Histology) and Bosch Institute, University of Sydney, Sydney, Australia

## Abstract

**Purpose:**

The damaging effects of oxidative stress and transforming growth factor-β (TGFβ)-induced transdifferentiation of lens epithelial cells have both been implicated independently in the etiology of cataract. The aim of this study was to investigate whether the presence of antioxidant systems in the lens influences the ability of lens epithelial cells to respond to TGFβ.

**Methods:**

Whole lenses from young rats were cultured with or without TGFβ in the presence or absence of reduced glutathione (GSH). Lens epithelial explants from weanling rats were used to investigate the effects of GSH and catalase on TGFβ-induced cataract-related changes. Lenses were monitored for opacification for three to four days, photographed, and then processed for routine histology. Explants were assessed by phase contrast microscopy, enzyme-linked immunosorbent assay (ELISA) of α-smooth muscle actin (αSMA), and/or immunolocalization of αSMA and Pax6, markers for transdifferentiation and normal lens epithelial phenotype, respectively.

**Results:**

In cultured lenses, GSH strongly suppressed TGFβ-induced opacification and subcapsular plaque formation. In explants, both GSH and catalase suppressed changes typically associated with TGFβ-induced transdifferentiation including wrinkling of the lens capsule, cell-surface blebbing, apoptotic cell loss, induction of αSMA, and loss of Pax6 expression.

**Conclusions:**

This study suggests that antioxidant systems present in the normal lens, which protect the epithelium against the damaging effects of reactive oxygen species, may also serve to protect it against the potentially cataractogenic effects of TGFβ. Taken together with other recent studies, it also raises the possibility that TGFβ may induce cataract-related changes in lens epithelial cells via release of hydrogen peroxide.

## Introduction

Cataract or loss of lens transparency is the major cause of blindness and visual impairment worldwide. Its prevalence is increasing as world populations age, resulting in escalating health costs and immense suffering [1-3]. Besides the well established association between aging and cataract, other predisposing factors include ultraviolet light exposure, smoking, diabetes, and steroid therapy [3,4]. The three major forms of cataract found in aging populations are nuclear cataract, cortical cataract, and posterior subcapsular cataract. While the incidence of posterior subcapsular cataract is lower than that of nuclear and cortical cataract, posterior subcapsular cataract has a greater effect on visual function, hastening the patient toward cataract surgery [1,4-6].

Oxidative stress leading to accumulation in the lens of reactive oxygen species (ROS) such as hydrogen peroxide (H_2_O_2_), the superoxide and hydroxyl radicals, and peroxynitrite is widely acknowledged to be a major initiating factor in the development of age-related cataracts [7-12]. Oxidative damage to the lens epithelium, which then spreads to the cortex, may lead to the formation of cortical cataract [7] while photo-oxidation of protein-bound kynurenine and its derivatives in the lens nucleus may be a significant event in the etiology of nuclear cataract [8]. In the latter type of cataract, insoluble aggregates of oxidized proteins accumulate in the nucleus of the lens. The normal lens is well supplied with primary antioxidants and several interconnected enzymic systems to protect it against reactive oxygen species [11].

The suggestion that transforming growth factor-β (TGF β) plays a role in the etiology of subcapsular cataract has been gaining increasing acceptance since its cataractous effects on lens epithelial explants were first reported in 1994 [13]. Subcapsular cataract is characterized by the presence of one or more opaque plaque(s) apposing the lens capsule in the posterior or anterior region of the lens. Features of these cataracts are myofibroblastic/fibroblastic transdifferentiation of lens cells and/or formation of aberrant swollen cells, abnormal migration and multilayering of cells, wrinkling of the lens capsule, and apoptotic cell death [14-20]. Intact rat lenses exposed to TGFβ in vitro and transgenic mice overexpressing active TGFβ in the lens develop opaque plaques beneath the anterior lens capsule that are strikingly similar to human anterior subcapsular cataracts [21-26]. The severity of the response to TGFβ in vitro increases with the aging of the animal [23].

Changes in lens epithelial cells induced by TGFβ include the induction of markers for epithelial-mesenchymal transdifferentiation such as α-smooth muscle actin (αSMA), types I and III collagen, and fibronectin; formation of myofibroblast-like spindle cells associated with wrinkling of the lens capsule; and loss of markers for lens epithelial phenotype such as Pax6 and E-cadherin (reviewed in [25,27]). TGFβ also induces apoptotic cell death accompanied by cell-surface blebbing and nuclear fragmentation [13,28-30]. These TGFβ-induced changes have been observed in studies of intact lenses, lens epithelial explants, and lens epithelial cell lines obtained from humans and other mammalian species spanning a wide range of ages. All three mammalian isoforms of TGFβ induce cataract-related changes, TGFβ2 and TGFβ3 being more potent than TGFβ1 [25]. TGFβ is present in the lens epithelium and in the aqueous that bathes the lens. However, its activity appears to be highly regulated under normal conditions [25,31,32].

TGFβ may influence the development of cataracts other than anterior subcapsular cataracts. In rat models in vivo and in vitro, TGFβ has been shown to induce the migration of transdifferentiated fibroblastic cells along the lens capsule toward the posterior pole [33,34], a common feature of posterior subcapsular cataract associated with aging, diabetes, and steroid use [17]. TGFβ also induced cortical changes analogous to those in human cortical cataract in these same studies. Very recently, a possible link between TGFβ and nuclear cataract has been identified [35]. Exposure of human lens epithelial cells to H_2_O_2_ in vitro triggers the formation of insoluble protein aggregates, which are typically present in nuclear cataracts. This occurs via a mechanism involving an H_2_O_2_-induced release of TGFβ followed by TGFβ/Smad signaling-dependent activation of transglutaminase 2, an enzyme that catalyzes protein cross-linking [35].

Oxidative stress-related damage and TGFβ-induced cataract-related changes are currently the focus of many investigations into age-related cataractogenesis. In the past, these two proposed mechanisms for cataractogenesis have been regarded as distinct and perhaps even mutually exclusive hypotheses. However, the emerging recognition that ROS may act as key intermediates in the signaling pathways of growth factors [36-39] suggests a possible nexus between them. To explore this, we used two well established rat models to investigate the effects of glutathione (GSH), an antioxidant, and catalase, a hydrogen peroxide (H_2_O_2_)-inactivating enzyme, on various TGFβ-induced cataract-related responses. GSH and catalase are both present in the normal lens where they serve to protect it against oxidative insult [11].

## Methods

Wistar rats were used for all experiments, and all procedures were in accordance with the ARVO Statement on the Use of Animals in Ophthalmic and Vision Research and approved by the Animal Ethics Committee of the University of Sydney (Sydney, Australia).

### Preparation and culture of whole lenses and lens epithelial explants

Whole lens cultures and lens epithelial explants were prepared from four- to five-week-old male rats and from 18- to 22-day-old rats, respectively, as described previously [21,40,41]. The medium used in all experiments (control medium) was serum-free medium M199 (Trace Biosciences, Sydney, Australia) supplemented with 0.1% BSA and antibiotics [23]. HEPES at a final concentration of 20 mM was added to the control medium during the initial pinning out of explants but not during subsequent culture. Lenses and explants were cultured at 37 °C in 5% CO_2_/air.

Whole lenses were cultured for three to four days with 0.3 or 1.75 ng/ml of human recombinant TGFβ2 (active form; R&D Systems, Minneapolis, MN) with or without the addition of reduced GSH (cell culture tested; Sigma, St. Louis, MO) at a final concentration of 10 mM. The medium was changed on day 2 of culture. In addition, some lenses were cultured with GSH alone or in the control medium only. GSH was dissolved in the control medium, neutralized with NaOH, and added 10 min before TGFβ on day 0. GSH was re-added on day 2 if present initially. Lenses were monitored and photographed during culture using a dissecting microscope (Wild, Heerbrug, Switzerland) adapted to dark field microscopy and then processed for routine histology [21].

Lens epithelial explants were precultured for three days to ensure the lens capsule was well covered (70%–100%) with a confluent monolayer of cells. The explants were then cultured for two days in the presence or absence of 10 mM GSH with or without 75 pg/ml TGFβ2, which was added 10 min after GSH. Morphological changes were monitored daily by phase contrast microscopy [40]. Explants were collected at the end of the culture period in 50 μl ice-cold distilled water and stored at −20 °C for the determination of αSMA and DNA.

In other experiments, freshly prepared explants were cultured for two to three days with 75 pg/ml TGFβ2 in the presence or absence of bovine liver catalase (cell culture tested; Sigma) at a final concentration of 300 units per ml (120 μg of protein per ml) as used by others [42,43]. Furthermore, to control for any non-specific protective effect due to the presence of additional protein in the medium, BSA at an equivalent protein concentration was added to all cultures that did not receive catalase. All explants were monitored daily by phase contrast microscopy. At the end of the culture period, they were stored as described above for the determination of αSMA and DNA or fixed as whole mounts and processed for immunolocalization of αSMA and Pax6 [27] using secondary antibodies conjugated with Alexa 488 and Cy3, respectively.

### α-Smooth muscle actin ELISA and DNA assay

After the addition of 50 μl of 10 mM ethylenediamine tetraacetic acid-0.02% Triton X-100, pH 10, explants were lysed by incubating for 30 min at 37 °C then cooled in ice. A sample of lysate was diluted with a carbonate buffer (15 mM Na_2_CO_3_-35 mM NaHCO_3_, pH 9.6, containing 0.0006% Triton X-100) and applied in quadruplicate (50 μl/well) to 96 well black MaxiSorp™ plates (Nalge Nunc International, Rochester, NY). Partially purified αSMA prepared from rabbit aorta [44,45] and diluted in carbonate buffer was used to set up a linear standard curve for each plate over a constant range (expressed in arbitrary units). After overnight incubation at 4 °C in a humidified environment, the plate was washed with phosphate-buffered saline (PBS), blocked with 100 μl/well casein blocking solution at 37 °C for 30 min, and rinsed with PBS-0.05% Tween-20. The casein blocking solution was prepared by dissolving 12.5 g of casein in 400 ml of 0.3 M NaOH with overnight stirring at 37 °C, adjusting to pH 7 with 5 M HCl, and adding 5 ml of 2% sodium azide and distilled water to a final volume of 500 ml. Mouse monoclonal anti-αSMA antibody (clone 1A4; mouse ascites fluid; Sigma) diluted in PBS-1% BSA (1:1,000) was applied (100 μl/well). The plate was incubated at 37 °C for 60 min and washed with PBS-Tween-20, and the bound antibody was detected using the QuantaBlu Fluorogenic Peroxidase Substrate Kit (Pierce, Rockford, IL). The horseradish peroxidase-conjugated anti-mouse immunoglobulin G supplied by the manufacturer was diluted with PBS-1% BSA (1:4,000) and applied for 60 min at 37 °C. The final substrate reaction was stopped after a 90 min incubation. Fluorescence was measured using a FLUOstar Galaxy plate reader (BMG Labtech, Offenburg, Germany). In addition, a sample of each lysate was diluted with distilled water for the determination of DNA using PicoGreen ds DNA Quantitation Reagent (Molecular Probes, Eugene OR) as previously described [21].

### Statistical analyses

GraphPad Prism software (La Jolla, CA) was used for all analyses. Quantitative data were analyzed by one-way ANOVA after log transformation with post hoc comparison of selected treatment groups and Bonferroni correction for multiple comparisons. The Mann–Whitney ranking test was used to assess differences in the opacification rankings of various treatment groups, and Fisher’s exact test was used to assess differences in the proportions of lenses or explants exhibiting a specific feature.

## Results

**Figure 1:**
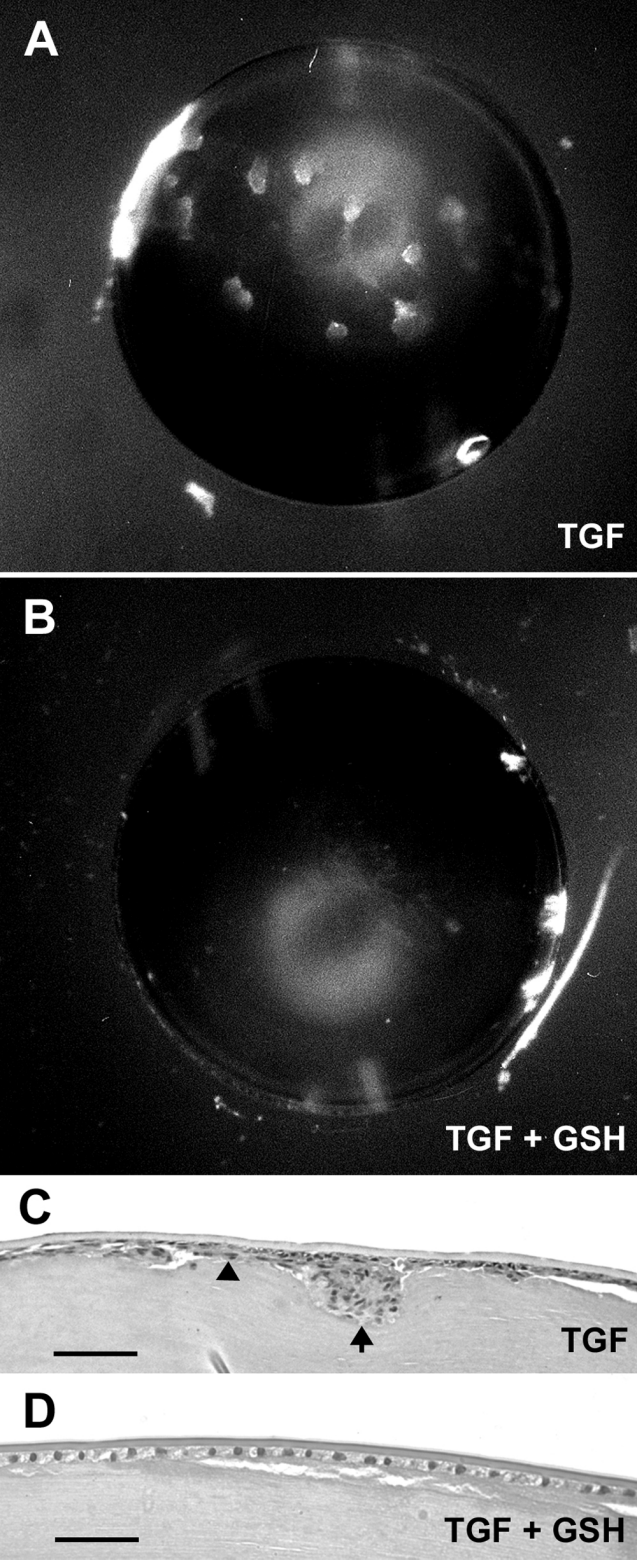
Suppression of TGFβ-induced lens opacification by GSH. **A**,**B**: Dark field microscopy. Lenses were cultured for three days with 1.75 ng/ml TGFβ2 in the absence (**A**) or presence (**B**) of 10 mM GSH and then photographed. Lenses cultured with TGFβ alone typically exhibited numerous discrete opacities (**A**) whereas many lenses cultured with TGFβ and GSH remained transparent (**B**). The crescent-shaped haziness in each image is an artifact of the light source. **C**,**D**: Hematoxylin-eosin stained lens sections. In lenses cultured with TGFβ alone (**C**), the epithelium typically became multilayered (arrowhead) and contained large anterior subcapsular plaques of cells (arrow). In the lens shown in **D**, which retained transparency during the four days of culture with 0.3 ng/ml TGFβ2 and GSH, the epithelium remained cuboidal and monolayered as it is in the normal lens. The scale bar represents 120 μm (**C**) and 60 μm (**D**). GSH, glutathione.

**Figure 2:**
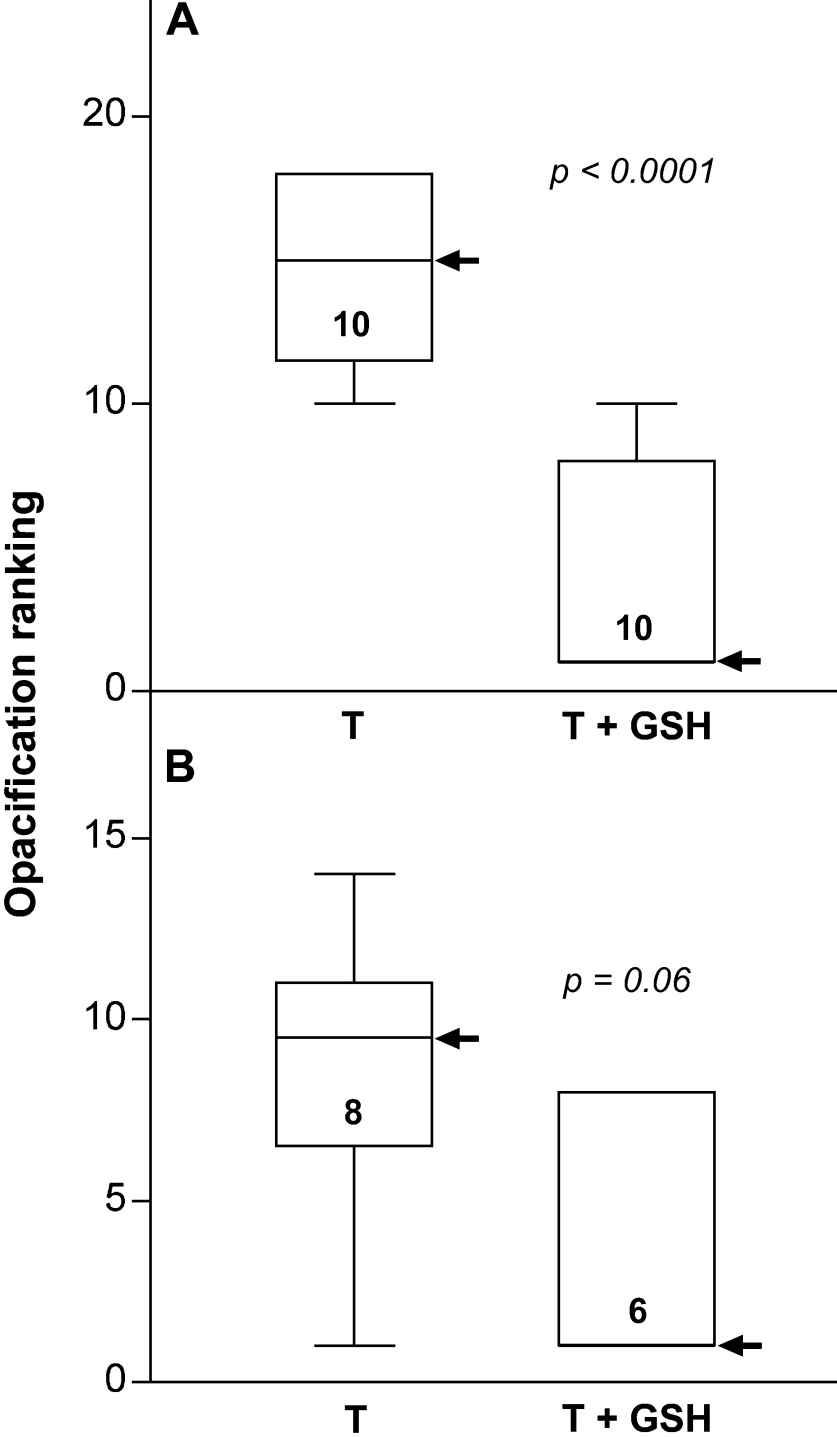
Suppression of TGFβ-induced lens opacification by GSH: statistical analysis. Lenses were cultured with TGFβ with or without the addition of 10 mM GSH. The concentration of TGFβ was 0.3 ng/ml (**A**) or 1.75 ng/ml (**B**). Photomicrographs recorded on day 4 (**A**) or day 3 (**B**) as illustrated in Figure 1 were coded and ranked according to increasing severity of the opacification response. Box and whiskers plots are shown with arrows indicating the median value. The numbers inside the boxes indicate the number of lenses assessed in each case, and the p values indicate the significance of the difference between the two treatment groups (Mann–Whitney test). GSH, glutathione; T, TGFβ.

Most lenses cultured with TGFβ2 developed overt opacities (Figure 1A) whereas those cultured in the control medium remained transparent as shown in previous studies [21,22]. The induction of opacities by TGFβ was strongly suppressed by including GSH in the medium (Figure 1B and Figure 2). By day 4 of culture, opacities were observed in 15 of a total of 18 lenses cultured with TGFβ whereas only 2 of 16 lenses developed opacities when GSH was included with TGFβ (p<0.0001). Histological assessment confirmed that TGFβ-induced lens opacification was associated with multilayering of the lens epithelium and the formation of deep plaques of abnormal cells beneath the anterior capsule (Figure 1C) as shown previously [21,22]. Lenses that remained transparent during culture with TGFβ and GSH either retained a monolayered epithelium typical of the normal lens (Figure 1D) or exhibited patchy multilayering of the epithelium without plaque formation (not shown). Lenses cultured in parallel with GSH alone remained transparent and showed no histological changes.

**Figure 3:**
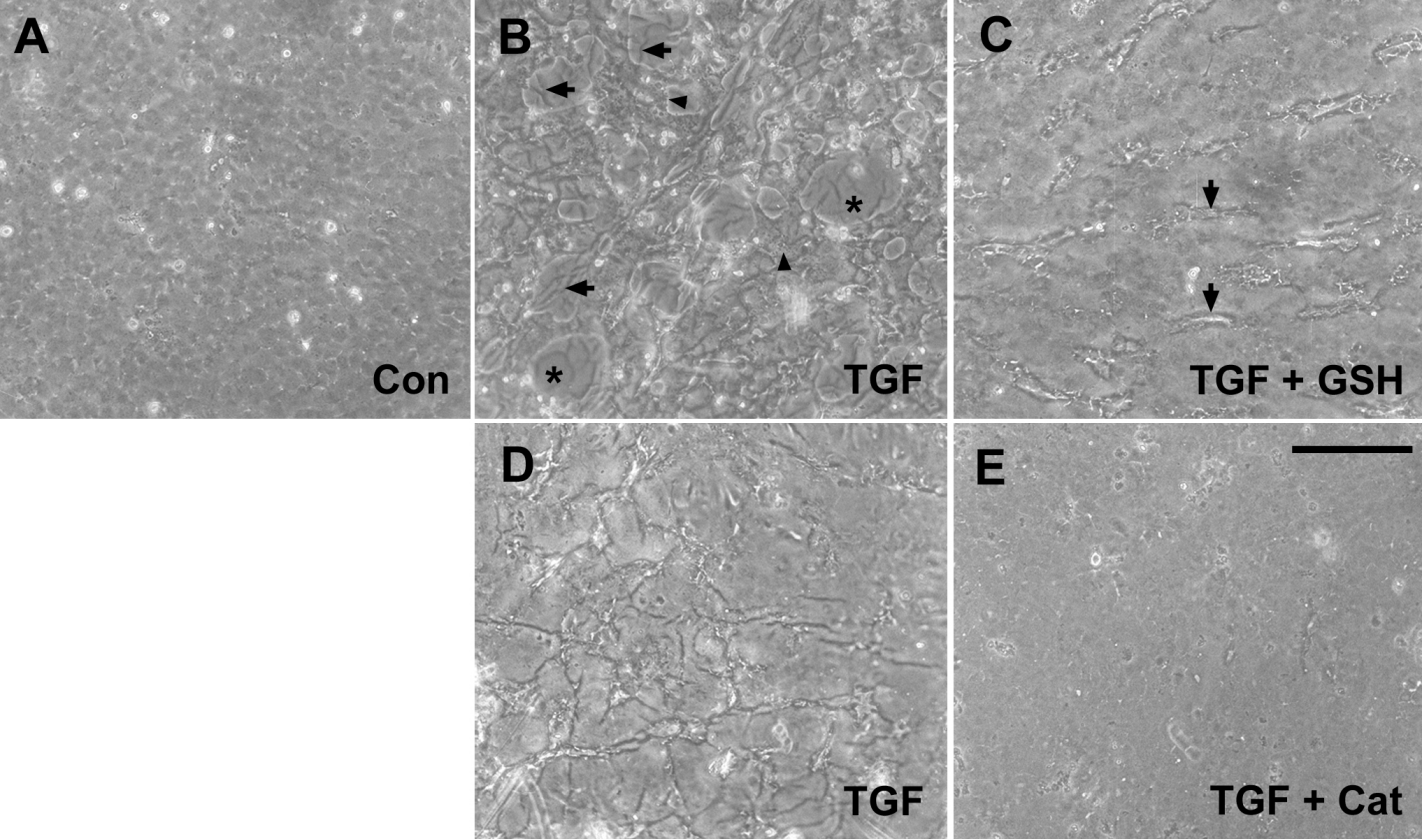
Suppression of TGFβ-induced cataractous changes in lens epithelial explants by GSH and catalase: phase contrast microscopy. **A-C**: Precultured explants were cultured for two days in the control medium (**A**) or with 75 pg/ml TGFβ2 in the absence (**B**) or presence (**C**) of 10 mM GSH and then photographed. In controls (**A**), the epithelial cells were present in the cobblestone array, typical of the normal lens epithelium. Explants cultured only with TGFβ (**B**) exhibited extensive wrinkling of the lens capsule (arrows) and loss of cells, leaving regions of the capsule exposed (asterisks). Many cells showed dark stippling indicative of blebbing of the cellular surface (arrowheads). Including GSH with TGFβ (**C**) suppressed these TGFβ-induced changes, resulting in slight wrinkling only (arrows). **D**,**E**: Explants were cultured for two days with 75 pg/ml TGFβ2 in the absence (**D**) or presence (**E**) of catalase at a concentration of 300 units/ml. Explants cultured with TGFβ alone were extensively wrinkled (**D**) whereas explants cultured with TGFβ and catalase retained normal cobblestone morphology (**E**). The scale bar represents 180 μm (**A-E**). Con, control; GSH, glutathione; Cat, catalase.

**Figure 4:**
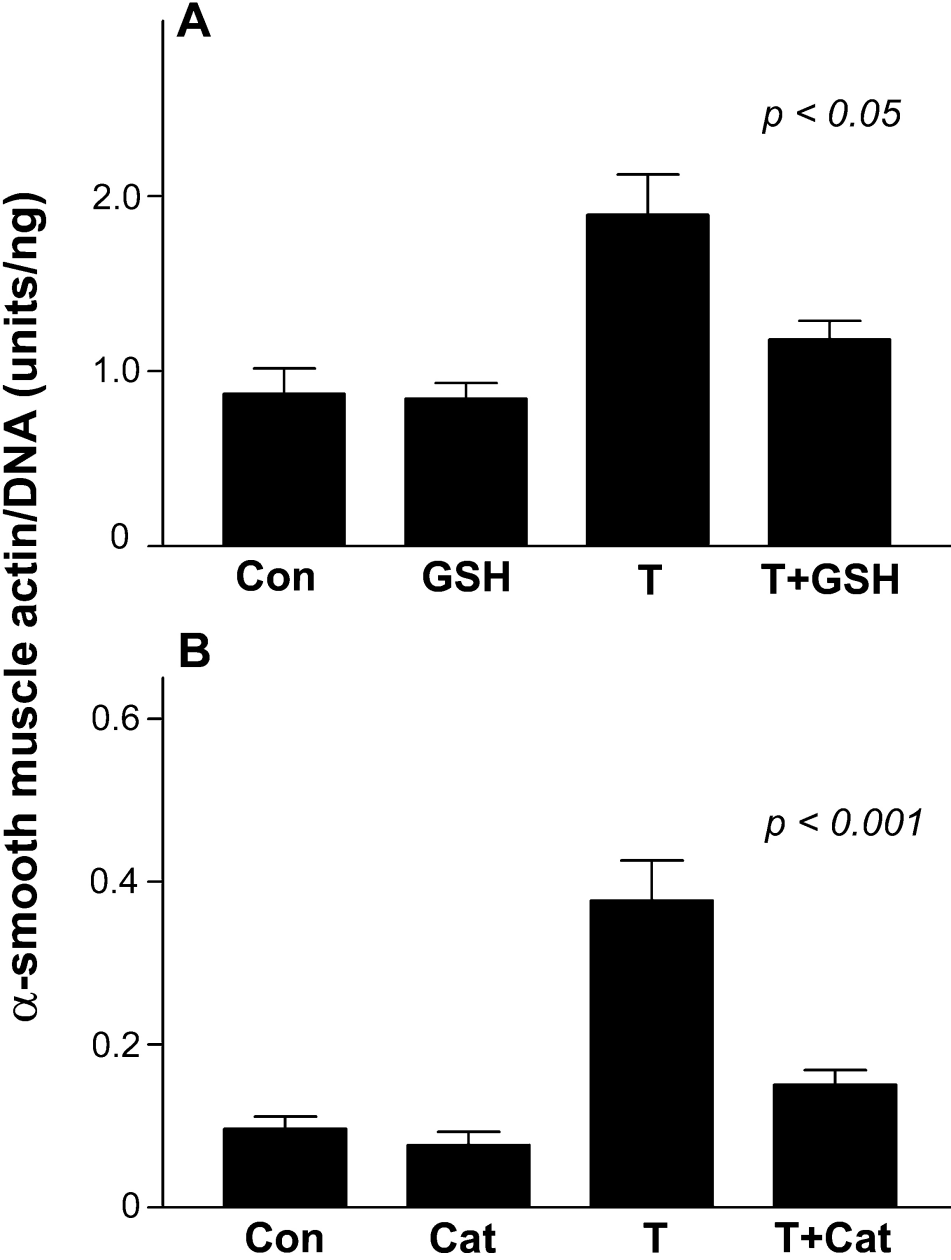
Suppression of TGFβ-induced αSMA expression by GSH and catalase: ELISA data. Explants were cultured in the control medium or with 75 pg/ml TGFβ2 in the absence or presence of 10 mM GSH (**A**) or 300 units/ml catalase (**B**). The culture period was 2 days (**A**) or 3 days (**B**). Explants were then lysed for determination of αSMA and DNA. Each value represents the mean±SEM, n=8–14 (**A**) and n=6–8 (**B**). In each case, the p value indicates the significance of the difference between the value for explants cultured with TGFβ alone and those cultured with TGFβ plus the test substance. Con, control; GSH, glutathione; T, TGFβ2; Cat, catalase.

We also assessed the effect of GSH on TGFβ-induced cataractous changes using rat lens epithelial explants. These explants undergo a range of distinctive morphological changes when cultured with TGFβ alone [13,40]. Extensive blebbing of the cellular surface, wrinkling of the lens capsule, and loss of cells induced by TGFβ are shown in Figure 3B (compare with control in Figure 3A). TGFβ also induced the formation of spindle-like cells in some explants, especially in the peripheral region (not shown). Including GSH with TGFβ markedly suppressed the TGFβ-induced morphological changes during the two-day culture period (Figure 3C). Extensive wrinkling was observed in 16 of 21 explants cultured with TGFβ alone compared with only 5 of 18 explants cultured with TGFβ and GSH (p=0.004). Furthermore, significant cell loss occurred in nine of the explants treated with TGFβ alone but in only one of the explants treated with both TGFβ and GSH (p=0.01). Induction of αSMA, the transdifferentiation marker, by TGFβ was also significantly inhibited by including GSH in the culture medium (Figure 4A).

The effect of catalase on TGFβ-induced cataractous changes was also investigated in explants. By day 2 of culture, suppression of TGFβ-induced morphological changes by catalase was evident (Figure 3D,E). By day 3 of culture, extensive cell surface blebbing was observed in six of eight explants cultured with TGFβ alone but only one of eight explants cultured with GSH and TGFβ (p= 0.04). In addition, obvious cell loss occurred in seven of the eight explants cultured with TGFβ alone but was not observed in the explants cultured with GSH and TGFβ (p=0.001).

**Figure 5:**
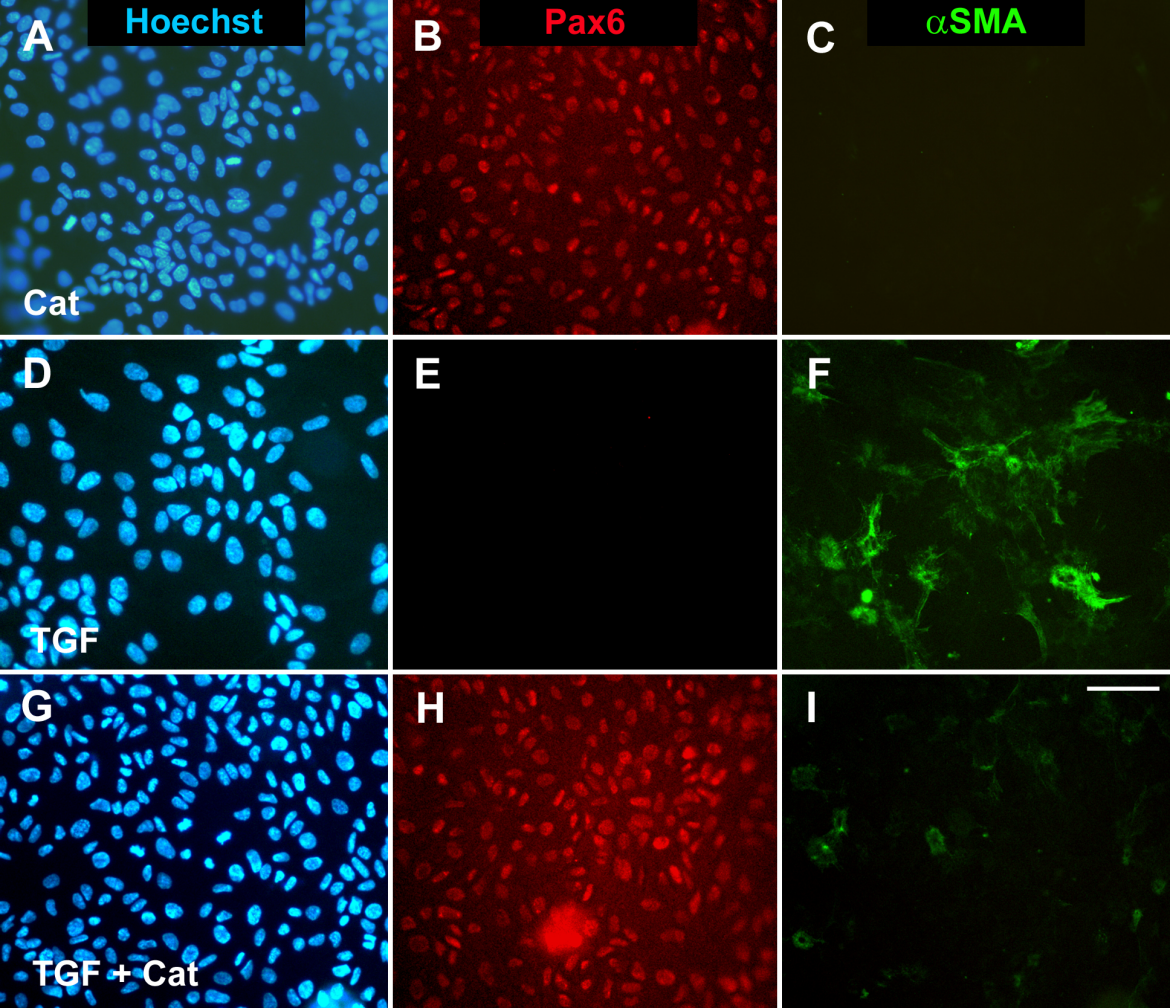
Suppression of TGFβ-induced changes in αSMA and Pax6 expression by catalase. Explants were cultured for two days with catalase alone at a concentration of 300 units/ml (**A-C**) or with 75 pg/ml TGFβ2 in the absence (**D-F**) or presence (**G-I**) of catalase and then fixed as whole mounts and processed for immunolocalization of Pax6 (red; **B**,**E**,**H**) and αSMA (green; **C**,**F**,**I**) by a double labeling technique. Nuclei were counterstained using Hoechst dye (blue; **A**,**D**,**G**). Images of the same explant region are shown in each row. Explants cultured with catalase alone were indistinguishable from the controls in that they exhibited strong nuclear expression of the lens epithelial cell marker, Pax6 (**B**), and lacked reactivity for the transdifferentiation marker, αSMA (**C**). Explants cultured with TGFβ lost reactivity for Pax6 (**E**) and contained many αSMA-positive cells (**F**). Catalase protected against TGFβ-induced loss of Pax6 expression (**H**) and and strongly suppressed the induction of αSMA by TGFβ (**I**). The scale bar represents 60 μm (**A-I**). Cat, catalase.

Cells in explants cultured with catalase alone consistently exhibited nuclear expression of Pax6 (Figure 5B), a marker for normal lens epithelial phenotype [25], as did cells in corresponding controls (not shown) and freshly prepared explants [41]. Culturing with TGFβ alone induced virtually complete loss of Pax6 (Figure 5E), and this loss was prevented by including catalase with TGFβ (Figure 5H). αSMA was not detectable by immunohistochemistry in explants cultured with catalase alone (Figure 5C) or in control explants (not shown). It was strongly induced by TGFβ (Figure 4B and Figure 5F), and including catalase significantly suppressed the induction of αSMA by TGFβ (Figure 4B and Figure 5I).

## Discussion

Oxidative stress, which has been implicated in the etiology of age-related cataract, occurs when the amounts of ROS generated in or near cells exceed the capacity of normal detoxification systems to deal with them. Cellular damage may arise due to the interaction of ROS with cellular constituents (see Introduction). The lens lacks blood vessels to assist in dispersing ROS, which are produced as a result of normal metabolic processes as well as under non-physiologic conditions. However, the lens is particularly well supplied with ROS detoxification systems such as the primary antioxidants, GSH and ascorbic acid, and enzymatic systems such as catalase, superoxide dismutase, and glutathione peroxidase, which maintain the normal adult lens in a highly reduced state [11]. GSH, normally present at high concentrations in the lens, provides the first line of defense against ROS [11]. GSH was used in the present study at a concentration estimated to be close to that of the normal adult rat lens cortex [11,46,47]. The concentration of GSH diminishes with aging especially in the nucleus of the lens, a change that may precede the development of age-related nuclear cataracts [8]. Besides acting as a general thiol antioxidant, GSH specifically removes H_2_O_2_ via the action of glutathione peroxidase. The ubiquitous enzyme catalase also avidly removes excess H_2_O_2_ [11].

Cataract-related changes induced by TGFβ may also play a role in cataractogenesis (see Introduction). Generally, the Smad pathway is regarded as the main mediator of TGFβ-signaling. Interaction of TGFβ with its receptor complex induces phosphorylation of Smad2/3, which upon linking with Smad4 translocate from the cytoplasm to the nucleus and influence gene expression [48]. However, in some cell types at least, other growth factors cooperate with TGFβ-dependent Smad signaling or act directly as mediators or modulators of TGFβ-dependent biological effects via Smad-independent signaling pathways such as the various mitogen-activated protein kinase cascades [48]. Three recent investigations indicate that signaling via Smad3 but not Smad2 plays a role in the induction of cataract-related changes by TGFβ [31,32,49]. However, in two of these studies, cataract-like responses to TGFβ were only partially suppressed in Smad3 knockout mice [31,49]. Moreover, TGFβ-induced apoptosis of lens epithelial cells, another feature of subcapsular cataracts (see Introduction), was shown to be significantly enhanced in Smad3 knockout mice [49]. These findings have led to the proposal that other TGFβ signaling pathways may be involved [31,49].

Here, we report results that raise the possibility that a signaling pathway that requires the release of H_2_O_2_ may contribute to the cataractogenic effects of TGFβ, which has been previously shown by others to be associated with Smad3 phosphorylation [31,32,49]. In particular, in the lens epithelial explant model, the H_2_O_2_-specific enzyme catalase suppressed changes typically associated with the TGFβ-induced transdifferentiation that is a hallmark of human subcapsular cataracts (see Introduction). These included wrinkling of the lens capsule, cell-surface blebbing, induction of αSMA, and loss of Pax6 expression. Catalase also suppressed TGFβ-induced loss of cells from explants, shown elsewhere to be the result of apoptotic cell death [13,30]. Moreover, the non-specific antioxidant GSH mimicked the effects of catalase in preventing TGFβ-induced cataract-like morphological changes in lens explants and strongly suppressed TGFβ’s ability to induce opaque, cataract-like subcapsular plaques in cultured lenses. Thus, the abundance of GSH and catalase in the lens may represent not only a defense against oxidative insult but also a regulatory mechanism for protecting the lens epithelium against the cataractogenic effects of TGFβ present in the lens environment in addition to mechanisms already recognized (see Introduction).

Recent studies of many different cell types have revealed a TGFβ-signaling pathway that is dependent upon the release of H_2_O_2_ with possible cross-talk between this new pathway and other signaling pathways [43,50-54]. Responses to TGFβ shown to be dependent upon the release of H_2_O_2_ in various non-lens cell types include proliferation; epithelial-mesenchymal transition and/or related upregulation of αSMA, collagen type I, and fibronectin [42,53,55-57]; downregulation of E-cadherin [53]; and induction of apoptosis [58,59]. Interestingly, it has been reported that Smad2 phosphorylation is dependent upon prior release of H_2_O_2_ by TGFβ in renal epithelial cells [53]. In other cell types, it has been shown that TGFβ induces different responses when acting via H_2_O_2_-dependent and H_2_O_2_-independent pathways, the concentration of TGFβ being a critical factor [58,60,61]. Production of H_2_O_2_, which generally occurs rapidly and transiently [54,59,62-64], requires the action of an NAD(P)H oxidase located on the plasma membrane [51,62]. Cellular responses to TGFβ mediated by H_2_O_2_ are inhibited by catalase, by antioxidants such as glutathione, N-acetylcysteine, and ascorbic acid, and by inhibiting NAD(P)H oxidase [42,43,51-59,62-65] or the type I TGFβ/ALK5 receptor [63]. Further, such responses to TGFβ are exacerbated by inhibiting GSH synthesis [56,59,65] and may be mimicked or enhanced by exposure of the cells to H_2_O_2_ [42,54,56-58,60].

The present study builds on previous studies in the lens. These previous studies provide evidence that TGFβ exerts at least some of its biological effects on lens cells via a ROS- or H_2_O_2_-dependent signaling pathway. Lenses of several mammalian species including the rat have been shown to contain the non-phagocytic NAD(P)H oxidase required for H_2_O_2_ release [11,39], and a rapid release of ROS by TGFβ has been demonstrated using human lens epithelial cell lines [39,61]. In one of the latter studies [61], apoptosis was induced by TGFβ only when present at a concentration that triggered ROS production. Under these conditions, apoptosis was inhibited by free radical scavengers, a result analogous to the finding reported here that glutathione suppressed morphological changes typically associated with TGFβ-induced apoptosis in lens epithelial explants. Furthermore, the ability of TGFβ to induce upregulation of 1-cysPrx mRNA and protein in human lens epithelial cells was mimicked by exposing the cells to a non-cytotoxic concentration of H_2_O_2_ [66]. The finding that the H_2_O_2_-specific enzyme catalase inhibited TGFβ-induced cataract-like changes in the present study strongly suggests a role for H_2_O_2_ in eliciting these responses, a suggestion that is supported by the results of the previous studies of lens epithelial cells and lenses that show a link between TGFβ stimulation and ROS or H_2_O_2_ release [39,61,66]. However, further investigation of the proposed involvement of H_2_O_2_ in TGFβ-induced cataract-like responses is warranted.

There have been numerous reports of the induction of cataract in cultured lenses exposed to H_2_O_2_ (for example, see [7,66,67] ). However, relatively high concentrations of H_2_O_2_ have often been used in such studies in excess of the cytotoxic range (100–200 μΜ) reported for lens epithelial cells [66-69], resulting in severe damage to the lens epithelium and rapid opacification, which may extend throughout the entire lens. It is not clear whether H_2_O_2_-mediated signaling contributes to the cataractogenesis observed under these conditions.

Our study taken together with other recent studies of the lens [35,39,61,66] indicates that future investigations of cataractogenesis will need to take into account not only the ability of oxidative stress or radiation-induced ROS release to adversely modify lens cells and their constituents by directly interacting with target molecules but also the possibility that H_2_O_2_ may serve as a signaling molecule in pathways that lead to cataract-related phenotypic changes in lens epithelial cells. Further, because active TGFβ is released from lens epithelial cells when the epithelium is damaged [31,70], it is possible that oxidative stress-associated cataractogenesis may be augmented by TGFβ-induced stimulation of  H_2_O_2_-dependent, Smad-dependent, and/or other growth factor signaling pathways. The possibility that H_2_O_2_ also mediates upregulation of TGFβ in the lens as in other cell types [53,57] remains to be investigated.

Thus, the present study highlights the need to approach future investigations of the etiology of cataract from a much broader, more holistic perspective, paving the way for novel experimental approaches to the study of cataractogenesis and its prevention. In addition, this study suggests that the complex array of enzymic and non-enzymic antioxidant systems present in the normal lens may serve not only to counter the direct assault of free radicals on lens cells and their constituents but also to protect the epithelium against the potentially cataractogenic effects of TGFβ.
